# Recent Approaches to the Identification of Novel Microtubule-Targeting Agents

**DOI:** 10.3389/fmolb.2022.841777

**Published:** 2022-03-30

**Authors:** Susanna Eli, Rossella Castagna, Marina Mapelli, Emilio Parisini

**Affiliations:** ^1^ IEO, European Institute of Oncology IRCCS, Milan, Italy; ^2^ Latvian Institute of Organic Synthesis, Aizkraukles Iela 21, Riga, Latvia

**Keywords:** tubulin drugs, microtubule drugs, chemotherapeutic agents, photopharmacology, photocaged, photoswitch, PROTAC, artificial intelligence

## Abstract

Microtubules are key components of the eukaryotic cytoskeleton with essential roles in cell division, intercellular transport, cell morphology, motility, and signal transduction. They are composed of protofilaments of heterodimers of α-tubulin and β-tubulin organized as rigid hollow cylinders that can assemble into large and dynamic intracellular structures. Consistent with their involvement in core cellular processes, affecting microtubule assembly results in cytotoxicity and cell death. For these reasons, microtubules are among the most important targets for the therapeutic treatment of several diseases, including cancer. The vast literature related to microtubule stabilizers and destabilizers has been reviewed extensively in recent years. Here we summarize recent experimental and computational approaches for the identification of novel tubulin modulators and delivery strategies. These include orphan small molecules, PROTACs as well as light-sensitive compounds that can be activated with high spatio-temporal accuracy and that represent promising tools for precision-targeted chemotherapy.

## Introduction

**FIGURE 1:**
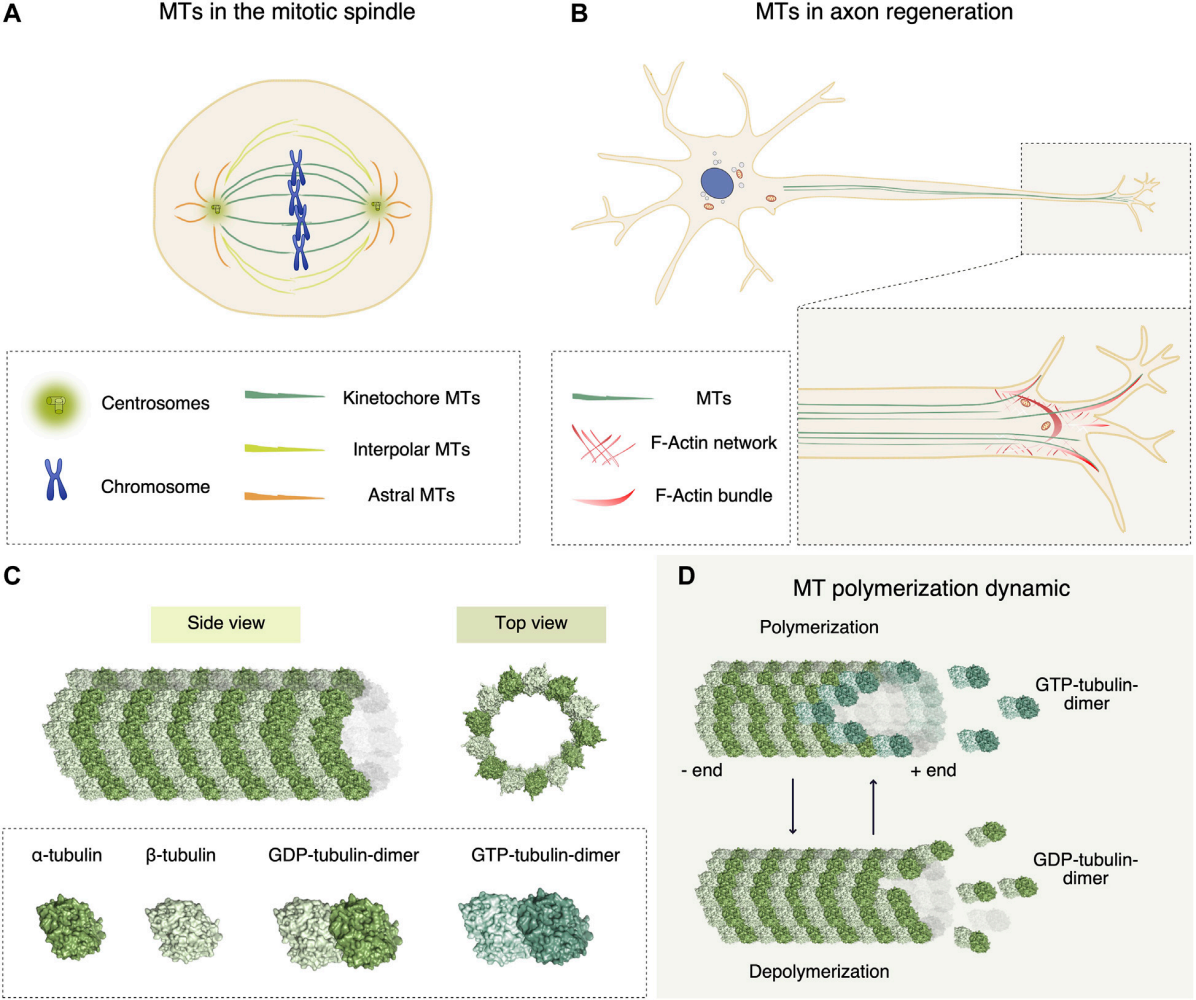
Functional roles of microtubules (MTs), and chemical molecules affecting their dynamic instability. **(A)** In mitosis, MTs contribute to the formation of the mitotic spindle, ensuring equal chromosomes segregation into two daughter cells. The mitotic spindle consists of kinetochore, interpolar and astral MTs that exert mechanical forces first to align the metaphase plate and then to divide sister chromatids equally. **(B)** In neural cells, the cytoskeleton dynamic generated by MTs and actin ensures the proper regeneration and polarization of axons. Bundled MTs are responsible for the elongation of axons, whereas in the growth cone cooperation of filopodial actin and polymerizing MTs leads to elongation. **(C)** MT protofilament architecture. α- and β-tubulin subunits assemble in a heterodimer (surface representation in light and dark green respectively, (PDB-ID 1JFF)), that form polarized protofilaments. MTs are hollow structures (PDB-ID 6O2T) formed by 13 protofilaments of α/β-tubulin dimers. **(D)** To elongate the protofilaments, during polymerization GTP-loaded tubulin dimers are added to the growing plus end of the MT, where the dynamics is more rapid compared to the minus end. During MT shrinking, GDP-loaded tubulin dimers dissociate from the lattice.

Microtubules (MTs) play a central role in many biological processes, ranging from cell signaling, cell morphology, to cell movement and division (Dustin, 1978). MTs organize networks that provide the structural scaffolding for vesicular trafficking and organelle positioning (Bonifacino and Neefjes, 2017) by acting as platforms for the motors kinesins and dyneins (Gennerich and Vale, 2009) (Figures 1A,B). In dividing cells, MTs directly contribute to the formation of the mitotic spindle ensuring equal chromosomes segregation in a highly orchestrated process (Rieder and Salmon, 1998).

The modulation of MT polymerization dynamics, also known as dynamic instability, is the major instrument that cells use to regulate all these biological functions. MTs are hollow structures formed by 13 polarized protofilaments of α/β-tubulin dimers (Figure 1C). MT polymerization consists in the addition of α/β-tubulin dimers at the MT ends (Figure 1D) (Waterman-Storer and Salmon, 1997), with rates in the order of minutes in interphase to seconds in mitosis (Saxton et al., 1984). A plethora of MT-binding proteins is crucial for the spatial and temporal regulation of MT dynamics. For this reason, proteins affecting MT organization have been studied as chemotherapeutic agents (Zhou and Giannakakou, 2005).

The first MT regulators have been identified in neurons (Cassimeris and Spittle, 2001). Indeed, the cytoskeleton dynamic generated by MTs and actin ensures the proper growth of neurons (Capizzi et al., 2021; Alushin et al., 2014) and polarization of axons (Blanquie and Bradke, 2018) (Figure 1B). The pharmacological modulation of cytoskeleton dynamics in injured axons may overturn a dystrophic structure to a regenerative one. For example, the MT stabilizing agent Taxol was proven to facilitate axonal regeneration after spinal cord injury by stimulating axonal growth and reducing fibrotic scarring (Hellal et al., 2011).

MT-targeting agents (MTAs) are valuable effectors for cancer therapy because they interfere with MT polymerization dynamics leading to mitotic arrest and apoptosis (Giannakakou et al., 2000). Many MTAs used in clinics were discovered by large-scale screening of natural compounds derived from bacteria, plants, fungi and sponges (Miller et al., 2018). Mimicking their function, synthetic inhibitors orchestrate MT filament dynamics by binding to tubulin according to their target binding site (Dumontet and Jordan, 2010). At high concentrations MTAs affect net MTs polymer mass, while at low doses they modulate dynamic instability (Wilson et al., 1999) causing a delay in mitotic entry or a mitotic block, with consequent induction of apoptosis (Steinmetz and Prota, 2018). Interestingly, many pieces of evidence correlate the lethality of MTAs with disruption of mitosis-independent functions such as cell signaling and vesicular trafficking (Komlodi-Pasztor et al., 2011). MTAs may also act synergically with DNA-damaging agents in cancer treatment by inhibiting the correct transport of DNA repair proteins into the nucleus (Poruchynsky et al., 2015). Thus, the molecular effects of each MTA may be very complex to dissect.

## MTAs and Their Use in the Clinics

**FIGURE 2:**
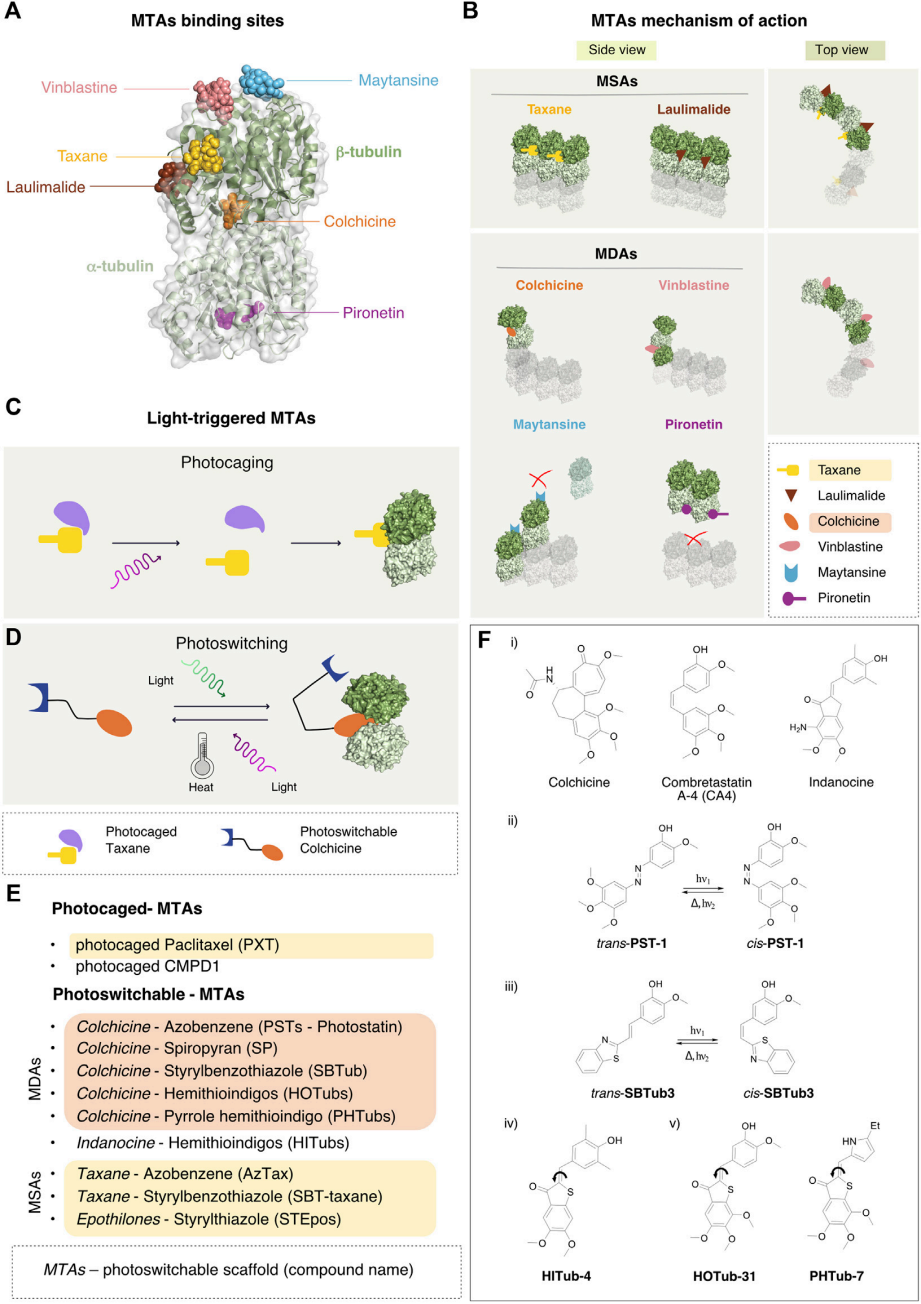
MTAs binding sites, mechanism of action and the photo-pharmacological approach. **(A)** Binding sites of the major classes of MT stabilizers and destabilizers. Cartoon and surface representation of the α/β-tubulin dimer with the six major sites targeted by MT binding molecules. Most molecules associate with pockets on the β-tubulin subunit, including maytansine (PDB-ID 4TV8, light blue), vinblastine (PDB-ID 5J2T, pink), the Taxane vincadermolide (PDB-ID 5LXT, gold), laulimalide (PDB-ID 4O4H, brown). Colchicine fits at the interface between α -tubulin and β -tubulin subunits of the hetero-dimer (PDB-ID 4O2B, orange). The only characterized MT destabilizer binding to α-tubulin is pironetin (PDB-ID 5LA6, purple). **(B)** Mechanism of action of the microtubule-binding agents. Diagram of the mechanisms of action of the MSAs Taxane (gold) and laulimalide (brown): these ligands bind between two adjacent β-tubulin subunits stabilizing the lateral interaction between protofilaments in the MT. In the MDAs category, colchicine (orange) inhibits MT growth by preventing the conformational change of the α/β-tubulin dimer required for MT-lattice formation. Vinca-site agents (pink) intercalate between two longitudinally-aligned α/β-tubulin dimers along the MT lattice leading to MT destabilization. Also maytansine agents (light blue) fit on top of the β-tubulin subunit, constraining longitudinal interaction between two α/β-tubulin dimers. Pironetin agents (purple) bind to the α-tubulin subunit and prevent its binding to the α/β-tubulin dimer in the same protofilament. **(C)** Activation of photocaged compounds. Prior to illumination, the pharmacologically active moiety of the drug is shielded by a photolabile element, which acts as a hindering functionality. Upon illumination, cleavage of the photolabile moiety occurs and the drug becomes irreversibly activated and primed for binding to the target. **(D)** A photoswitchable element in the scaffold of the drug allows a reversible conformational change upon illumination with a specific wavelength of light, thus causing drug activation. The back reaction can either occur spontaneously upon light cessation (thermal activation), or by illumination with a different wavelength of light. **(E)** Existing photoswitchable versions of known MTAs. The name of the original MTA drug is indicated first, followed by the type of scaffold used for the synthesis of the photoswitchable analog and by the name of the corresponding photoswitchable MTA thus obtained. **(F)** Selected examples of microtubule-targeting agents. i) Lead structures of selected microtubule-destabilizing agents used for the development of photoswitchable analogues. ii) *Trans-cis* photoisomerization reaction of photostatin (PST-1) iii) *Trans-cis* photoisomerization reaction of styrylbenzothiazole (SBTub3) iv) Chemical structure of a hemithioindigo-based indanone-like tubulin inhibitor (HITub-4) v) Chemical structure of a hemithioindigo colchicinoid tubulin inhibitor (HOTub-31) and a pyrrole hemithioindigo tubulin inhibitor (PHTub-7).

MTAs are usually classified in two groups, according to their mechanism of action: the microtubule-stabilizing agents (MSAs), which promote their assembly (Dumontet and Jordan, 2010), and the microtubule-destabilizing agents (MDAs), which trigger MTs disassembly in α/β-tubulin oligomers or dimers. The specific binding site and the corresponding mechanism of action of each compound was identified and investigated at the atomic level by crystallographic studies (for a review see (Steinmetz and Prota, 2018)), recently integrated with cryo-EM analysis of entire MT filaments (Alushin et al., 2014). Six different MTA binding sites have been characterized on the α/β-tubulin dimer. The β-tubulin binding sites are the Taxane, Laumalide, Colchicine, Vinca and Maytansine site. The Pironetin site is the only one located on the α-tubulin subunit (Usui et al., 2004) (Figure 2A). In the following paragraphs, we will survey the different MTAs, their binding site, mechanism of action and current clinical applications.

### Microtubule-Stabilizing Agents—MSAs

#### Taxane-Site

Since their discovery in the seventies (Wani et al., 1971), Taxanes have been studied extensively. This category includes first-generation anticancer drugs, such as paclitaxel and docetaxel, and new generation molecules, as cabazitaxel that challenge drug resistance (de Bono et al., 2010). The cause of Taxane-resistance has been identified in the overexpression of class III β-tubulin (Mozzetti et al., 2005) and in its affinity for multidrug-resistance proteins, such as P-glycoprotein (Goldstein, 1996). Taxanes bind to β-tubulin in a MT luminal side pocket with polar and hydrophobic interactions, resulting in non-functional MTs that prevent cells from proceeding into anaphase (Figure 2B). They promote MT polymerization by binding to tubulin filaments with particularly high affinity for regions that are relevant for inter-protofilament contacts. Taxanes are widely used for the treatment of ovarian, breast and lung cancer (Ojima et al., 2015). To reduce the toxicity derived from formulations needed to solubilize paclitaxel, the use of nanoparticles of albumin-bound Taxanes has been studied (Stinchcombe, 2007). In addition, Taxanes conjugated with tumor-targeting antibodies (Guillemard and Saragovi, 2001) or vitamins (Russell-Jones et al., 2004) are under development in order to redirect the effect of the drug specifically to the cancer stem cells. Local delivery of Taxol may enhance also the regenerative capacity of injured neurons by inducing efficient axon regrowth in the optic nerve (Sengottuvel et al., 2011).

#### Laulimalide and Peloruside-A

Laulimalide and Peloruside-A are part of the MT-stabilizing family. They bind to β-tubulin, facing the lateral interface between tubulin protofilaments, thus inhibiting their disassembly (Figure 2B). These compounds act via two mechanisms: they inhibit interactions between α/β-tubulin dimers and at the same time they strengthen the lateral contacts between protofilaments by stabilizing the Taxane-site M-loop (Prota et al., 2014b). This crosstalk opens the possibility for combinatorial strategies of Laulimalide/Peloruside with Taxane-site ligands (Khrapunovich-Baine et al., 2011). Interestingly, Laulimalide and Peloruside were found effective against paclitaxel multidrug-resistant cancer cell lines with mutations in the Taxane site and overexpression of P-glycoprotein (Liu et al., 2007).

### Microtubule-Destabilizing Agents - MDAs

#### Colchicine-Site

Colchicine was the first MT-destabilizing agent to be discovered (Borisy and Taylor, 1967). It strongly impairs mitotic progression but its use in clinics is limited due to its high toxicity and low therapeutic index. Since the structural characterization of its binding mode (Ravelli et al., 2004), many colchicine-binding site inhibitors (CBSIs) have been developed as anti-inflammatory agents that primarily prevent the activation of the inflammatory bodies (Gasparyan et al., 2015). The binding pocket of these compound is located in the intermediate domain near the α/β−dimer interface (Figures 2A,B). When MTs assemble, α/β-tubulin dimers alter their architecture from a curved to a straight conformation (Nogales et al., 1998). Inhibitors of the colchicine family prevent this conformational change. Podophyllotoxin and Combretastatin bind in the same pocket as colchicine and are past phase II in different clinical trials (Young and Chaplin, 2004). Nocodazole is a reversible inhibitor of this pocket widely used in research as synchronization and anti-mitotic agent.

#### Vinca-Site

Compounds that target the Vinca site comprise different molecules of both natural and synthetic origin that destabilize MTs. They include the Vinca alkaloids, vinblastine, vincristine, eribulin, diazonamides and trizolopyrimidies (Moudi et al., 2013), which all bind between two α/β-tubulin dimers, at the longitudinal interface of protofilaments. Their binding site consist in a core pocket extending with additional contacts toward the GTP-binding site of β-tubulin at the plus end of the filament (Ranaivoson et al., 2012) (Figure 2C). Vinca-site compounds hamper the conformational changes of α/β-tubulin dimers necessary for their correct incorporation into MT filaments, and sequester them in ring-like oligomers incompatible with the straight protofilaments lattice (Na and Timasheff, 1980). In addition, Vinca drugs inhibit the hydrolysis of exchangeable GTP, bound to β−tubulin (David-Pfeuty et al., 1979). The working principles of these agents highlight the importance of the correct longitudinal curvature of the α/β-tubulin dimers for MT dynamics, as an additional possible target for further destabilizing mechanisms. Vinca alkaloids were first used in the treatment of childhood hematologic malignancies and after for solid and adult hematologic malignancies (Bohannon et al., 1963). Many semisynthetic analogs as vindesine (Gökbuget and Hoelzer, 1997), vinorelbine and vinflunine, were developed to overcome side effects such as neuropathy and myelosuppression, likely linked to altered axonal transport.

#### Maytansine-Site

Maytansine binds on the exposed side of the β-tubulin subunit (Prota et al., 2014a). It works by blocking the longitudinal protofilament interaction by either inhibiting the addition of dimers or by the formation of unfunctional complexes with β-tubulin (Figures 2A,B). Differently from Vinca alkaloids, maytansine prevents the correct turn-over of GTP-loaded β-subunits at the MT minus-ends (Lopus et al., 2010). Many companies are focusing on the development of antibody-maytansinoids conjugate (ADC) therapeutics, which suppress MT dynamic instability in mitosis (Oroudjev et al., 2010). Maytansine derivates are currently in use for metastatic breast cancer (Krop et al., 2012).

#### Pironetin-Site

Pironetin and its dimethyl derivatives destabilize MT polymerization and preclude mitotic progression. These ligands fit into a hydrophobic pocket in the α-tubulin subunit, adjacent to the α/β-tubulin dimers interface (Yang et al., 2016) (Figures 2A,B). In this way they promote the disassembly of the MT protofilaments by meddling the longitudinal contacts between dimers. Because of their peculiar binding-site, these compounds may be effective for the treatments of tumors with resistance against β-tubulin-targeting drugs.

Lately, some innovative approaches have provided interesting results and indicated possible ways forward in the discovery of new MTAs. Here, we will briefly review some of the most recent developments in the field.

## Photopharmacology: Light-Triggered Analogues of MTAs

The use of photosensitive drugs, which are activated by specific wavelengths of light, can provide good spatio-temporal control over drug action (Hoorens and Szymanski, 2018; Matera et al., 2018; Fuchter, 2020). Although still at the preclinical stage, photopharmacology may indeed help overcome crucial pharmacological problems in the future and provide alternative therapeutic strategies. Interestingly, existing clinically approved photodynamic therapies, which are based on the use of light in combination with photosensitizing agents to promote the formation of cytotoxic reactive oxygen species (ROS), are considered less-invasive than traditional pharmacological approaches. As such, they are widely investigated for cancer treatment (Huang, 2005), although they are occasionally affected by such limitations as inefficient deep-tissue penetration, incomplete tumor suppression and the insurgence of side effects from the patient’s accidental exposure to strong Sun/indoor light after treatment.

The design of both light-activated compounds and photopharmacological agents for targeted cancer therapies has recently been extensively reviewed with respect to their clinical potential (Vickerman et al., 2021) as well as their use in photodynamic therapy (Zhao et al., 2021) and in photopharmacology (Dunkel and Ilaš, 2021). There are two different types of light-triggerable bioactive compounds: photocaged and photoswitchable. The former are molecules in which the pharmacologically active moiety is shielded by a photolabile element that can be cleaved upon illumination, thus activating the drug irreversibly (Figure 2C), while the latter are drugs whose chemical scaffold accommodates a photoswitchable element, whereby the conformation and activity of the drug can be controlled in a reversible manner using light (Figure 2D).

### Photocaged Compounds

In photocaged compounds, the covalent attachment of the photolabile element in the starting form of the drug renders the compound inactive. However, when the hindering functionality is cleaved by irradiation with a specific wavelength of light, the drug is primed for binding to its target and exerts its inhibitory function. This activation process, albeit irreversible, allows a good degree of spatio-temporal control of the drug.

The first light-triggered microtubule-targeting agent ever developed was a photocaged version of paclitaxel, activated by nitrogen-pulsed laser in the ultraviolet (UV wavelength at 337 nm) (Buck and Zheng, 2002). Later, to improve cleavage efficiency and to obtain a completely inactive caged paclitaxel that could exert its activity exclusively upon illumination (UV-activated at 360 nm), a double caging strategy was developed (Gropeanu et al., 2012).

A visible-light-activated (430 nm) caged paclitaxel was subsequently developed using a 7-N,N-diethylamino-4-hydrozymethyl coumarin photolabile group (Skwarczynski et al., 2006). Further optimization of this class of photolabile-paclitaxel (phototaxel, UV-activated at 355 nm) led to compounds that feature greater water solubility (>100 mg/ml) and stability in physiological conditions (Noguchi et al., 2008).

More recently, a photocaged version of the small molecule CMPD1, another tubulin polymerization inhibitor, was synthetized using 4,5-dimethoxy-2-nitrobenzyl (DMNB) as photolabile protecting group (UV-activated at 365 nm). The tubulin inhibitor thus released was shown to be toxic for glioblastoma cells and to lead to apoptotic cell death (Döbber et al., 2017).

### Photoswitchable Compounds

Photoswitchable compounds undergo a conformational change when illuminated by proper wavelengths of light in the UV-vis spectra. However, upon switching conformation, the molecule becomes thermodynamically less stable and the compound is converted back to its thermally stable geometry. This can occur either by spontaneous back reaction on a time scale that ranges from fractions of a second to years, or by illumination with different wavelengths of light.

Photoswitchable drugs are typically designed via a bioisosteric replacement approach, whereby an azobenzene moiety is introduced in the chemical structure of the starting drug to act as photoswitchable unit. This *azologization* process is usually considered a mildly invasive chemical change in the drug scaffold. Alternatively, the azobenzene moiety can be attached to the drug in a lateral position and this approach is called *azoextension*. The aim of both approaches is to favor or to disturb the pharmacological activity of the compound by switching its conformation between the two isomers. Ideally, the goal is to obtain a compound that is pharmacologically inactive in the *trans* form and becomes activated by switching to the *cis* conformation. By implementing an *azologization* strategy on the structure of combretastatin A-4 (CA4), one of the most prominent colchicine binding site inhibitors, Borowiak et al. developed a class of photoswitchable analogs of CA4 named photostatin (PSTs), which are 250 times more active in their *cis* form (obtained by illumination in the range 370–430 nm) than in their *trans* conformation (Borowiak et al., 2015). Further *in vitro* (Engdahl et al., 2015) and *in vivo* (Zenker et al., 2017) studies have confirmed that PSTs are a robust chemical tool for exerting a photoswitchable cytotoxic effect with excellent optical control over microtubule dynamics at the single cell level.

The success of the photoswitchable design of PSTs relies on the structural features of CA4, a stilbenoid molecule that is well suited for an *azologization* approach. Indeed, unlike other CA4 analogues, it retains solubility and biological activity over tubulin upon *azologization* (Tron et al., 2006; Gosslau et al., 2008; Rastogi et al., 2018). Moreover, CA4 binds to the colchicine binding site in a *cis*-like conformation (PDB 5LYJ), which was shown to provide greater stabilization energy than the trans form (Gaspari et al., 2017).

Incidentally, azobenzene is not the only scaffold used in photopharmacology. The styrylbenzothiazole (SBT) moiety has been recently used as a scaffold for the design of photoswitchable microtubule inhibitors (Gao et al., 2021b). These compounds have been shown to be metabolically stable and to allow the photocontrol of microtubule dynamics with sub-cellular precision, photoswitched either by one-photon excitation (LED at 360 nm), or by two-photon excitation (laser at 780 nm). Moreover, they bind to the colchicine binding site in a cis-like conformation, similarly to CA4, as shown in the crystal structures of Z-SBTub2 (PDB: 6ZWC) and Z-SBTub3 (PDB: 6ZWB) bound to the tubulin-DARPin D1 complex.

The fact that the patented tubulin-binding anti-cancer drugs are structurally extremely diverse (Haider et al., 2019) provides many interesting opportunities for photopharmacology. Hemithioindigos (HTI) have been used as photoswitches in the design of microtubule binders based on the colchinoid pharmacophore. These compounds, which are cell-compatible and show antimitotic photoswitchable bioactivity, have been named hemithioindigo-colchicinoid tubulin binders (HOTubs, 450 nm) (Sailer et al., 2019). Likewise, the microtubule inhibitor colchicine served as template for the design of pyrrole hemithioindigo (PHTubs) compounds (photoswitched in the range 435–450 nm) (Sailer et al., 2021). Furthermore, colchicine has been used as structural reference for the development of photoswitchable cytotoxic compounds based on spiropyran (SP, exposed to UV light at 365 nm) (Rastogi et al., 2021).

By further optimization of the HTI scaffold, a photoswitchable class of compounds based on tubulin-inhibiting indanones have also been proposed (Sailer et al., 2020). The hemithioindigo-based indanone-like tubulin inhibitors (HITubs) show improved cellular potency relative to HOTubs as well as antimitotic photo-modulated activity (photoswitched with visible light at 450 and 530 nm).

Besides the generation of photoswitchable versions of microtubule destabilizers based on colchicine analogues, the photopharmacology community has also focused on the development of photoswitchable versions of microtubule stabilizers compounds (Figures 2E,F). A photoswitchable version of the microtubule stabilizing drug paclitaxel has been developed using an *azoextension* approach. The 3′-azobenzamide-Taxanes (AzTax) series of compounds proposed allowed the spatio-temporal control of microtubule stabilization in living cells (photoswitched in the range 360–530 nm) (Müller-Deku et al., 2020).

The possibility to use the styrylbenzothiazole (SBT) scaffold to obtain an *SBT-extension* of the Taxane drug was recently investigated. Unfortunately, the SBT-Taxane (SBTax) compounds show poor solubility, low bioactivity and modest photocontrol on the biological process. The *extension* approach has been also applied with styrylthiazole (ST) to epothilones (STEpos, photoactivated at the 405 nm laser line), which are a class of MSA targeting the same Taxane binding site (Gao et al., 2021a).

Recently, a rationalization of the protocol for handling photosensitive drugs to photocontrol microtubule dynamics in biological assays has been proposed (Thorn-Seshold and Meiring, 2021).

## PROTACs

PROteolysis TArgeting Chimeras (PROTACs) is a technology that holds great promise for overcoming drug resistance problems as it allows the inactivation of the target protein by inducing its complete degradation rather than its sheer inhibition (Garber, 2021). PROTACs are bifunctional molecules featuring an E3 ubiquitin ligase moiety tethered to a ligand of the target protein of interest via a linker of optimal length. The association of the ligand moiety of the PROTAC with the protein of interest promotes ubiquitination of the target protein and its degradation by the ubiquitin proteasome system (UPS). Recently, an attempt to develop the first tubulin-targeting PROTAC has been made (Gasic et al., 2020). The validity of this approach is corroborated by the observation that a number of compounds that are known to bind covalently to different cysteines on β-tubulin promote tubulin degradation (Yang et al., 2019). The authors designed different degrader molecules, all of them featuring a E3 ubiquitin ligase Cereblon (CRBN) moiety and either a monomethyl auristatin E (MMAE) scaffold or a combretastatin A-4 (CA4) scaffold as tubulin-binding moiety, the former binding at the interface between α and β-tubulin, the latter binding only to β-tubulin. However, neither strategies resulted in tubulin degradation. While the authors conclude that tubulin may be resistant to degradation by CRBN-recruiting PROTACs, they also suggest that the use of other E3 ligases or the use of a different tubulin-binding moiety may eventually lead to a successful tubulin degradation by different PROTACs. It is also conceivable that by combining photoactivation with protein degradation, the use of PHOTACs (PHOtochemically TArgeting Chimeras) may enable a precise spatio-temporal control of degraders by light (Reynders et al., 2020) and could represent a promising approach to high precision modulation of microtubule stability.

## Artificial Intelligence

Artificial intelligence has established itself as the most effective way to explore the chemical space in drug discovery. Recently, a drug-target prediction platform called BANDIT, which allows the integration of different chemical, genomic, clinical and pharmacological data types in a single multi-dimensional screening approach has been developed and used successfully for the identification of novel tubulin interactors (Madhukar et al., 2019). BANDIT integrates more than 20 million data points from such diverse data types as drug efficacy, post-treatment transcriptional responses, drug structures, reported adverse effects, bioassay results, and known targets. This integrated approach allows the identification of drugs that share the same target much more accurately than approaches that use single data types. Using this approach on microtubules, the authors initially identified a set of 24 structurally diverse orphan small molecules, which they tested experimentally on breast cancer cells. Of these 24 compounds, 14 were active on microtubules. Interestingly, only nine of these 14 compounds were also identified using structure-based only prediction methods. Tested on an ovarian carcinoma cell line that is resistant to Eribulin, an FDA approved MDAs, the authors identified three compounds that show good microtubule depolymerization activity against these cells, thus overcoming the Eribulin resistance problem.

## Conclusion

Because of its essential role in mitosis, tubulin is a fundamental target in drug discovery and an important benchmark for testing new cancer therapeutic approaches and ideas. This continuous quest for novel microtubule interactors is also justified by the drug resistance problem, which is often hampering the clinical efficacy of the current gold standard MTAs such as paclitaxel and vinblastine. Here we reported some of the most interesting and innovative recent approaches to MT modulation. It is likely that some of them will continue to be explored to identify new MTAs that may eventually provide viable alternatives to the current therapeutic protocols.
